# Metformin inhibits 17β-estradiol-induced epithelial-to-mesenchymal transition *via* βKlotho-related ERK1/2 signaling and AMPKα signaling in endometrial adenocarcinoma cells

**DOI:** 10.18632/oncotarget.7040

**Published:** 2016-01-27

**Authors:** Zhao Liu, Shasha Qi, Xingbo Zhao, Mingjiang Li, Sentai Ding, Jiaju Lu, Hui Zhang

**Affiliations:** ^1^ Department of Urology, Shandong Provincial Hospital Affiliated to Shandong University, Jinan, Shandong, People's Republic of China; ^2^ Department of Obstetrics and Gynecology, Shandong Provincial Hospital Affiliated to Shandong University, Jinan, Shandong, People's Republic of China

**Keywords:** metformin, βKlotho, 17β-estradiol, epithelial-mesenchymal transition, endometrial adenocarcinoma

## Abstract

The potential role of metformin in treating endometrial cancer remains to be explored. The current study investigated the role of metformin in 17β-estradiol-induced epithelial-mesenchymal transition (EMT) in endometrial adenocarcinoma cells. We found that 17β-estradiol promoted proliferation and migration, attenuated apoptosis in both estrogen receptor (ER) positive and ER negative endometrial adenocarcinoma cells (Ishikawa and KLE cells, respectively). Metformin abolished 17β-estradiol-induced cell proliferation and reversed 17β-estradiol-induced EMT in Ishikawa cells. In addition, metformin increased the expression of βKlotho, a fibroblast growth factors (FGFs) coreceptor, and decreased ERK1/2 phosphorylation in both Ishikawa and KLE cells. Decreased expression of βKlotho was noted in human endometrial adenocarcinomas, and plasmid-driven expression of βKlotho in Ishikawa cells abolished 17β-estradiol-induced EMT via inhibiting ERK1/2 signaling. βKlotho expression and metformin show synergetic effects on the proliferation and the EMT in Ishikawa cells. Furthermore, we demonstrated that the anti-EMT effects of metformin could be partly abolished by introducing Compound C, a specific AMPKα signaling inhibitor. In conclusion, metformin abolishes 17β-estradiol-induced cell proliferation and EMT in endometrial adenocarcinoma cells by upregulating βKlotho expression, inhibiting ERK1/2 signaling, and activating AMPKα signaling. Our study provides novel mechanistic insight into the anti-tumor effects of metformin.

## INTRODUCTION

Endometrial adenocarcinoma is the most common gynecological cancer worldwide [1], and one-half of patients died for advanced disease [2]. A better understanding of mechanism underlying the progress of endometrial carcinoma is required to facilitate the development of effective therapeutic strategies.

A series of reports indicate that the epithelial-mesenchymal transition (EMT) plays an important role in the tumorigenesis, progression, and chemoresistance of multiple carcinomas [3-5], including endometrial carcinoma [6]. During the EMT, epithelial cells undergo extensive alterations in gene expression, resulting in the loss of apical-basal polarity, the severing of intercellular adhesive junctions, and the degradation of basement membrane components [7]. In this way, they become mesenchymal cells with the characteristics of increased migration and invasion. The loss of E-cadherin is generally accepted as a hallmark of the EMT [8], which reduces cell-cell adhesion and destabilizes the epithelial architecture. This process is accompanied by increased expression of mesenchymal-related proteins, including N-cadherin, Vimentin, and fibronectin, which bestow a motile phenotype on cancer cells through changes in cellular architecture and cell-matrix interactions [9, 10]. Many transcription factors, such as Snail and Slug, act as repressors of E-cadherin in response to TGF-β [11] and IGF-IR [12] signaling, and have been linked to the induction of the EMT under different cellular contexts. In endometrial carcinoma, alterations of EMT-related markers have been associated with metastatic disease and reduced survival [13, 14]. Although the EMT has been broadly described in endometrial carcinoma, the molecular pathways involved are still poorly delineated.

As a hormone-dependent disease, endometrial carcinomas are sensitive to endogenous and exogenous estrogens, which are known risk factors for the disease. Recent studies showed that the 17β-estradiol/estrogen receptor (ER) signaling pathway is involved in the EMT in many carcinomas, including ovarian cancer [15], breast cancer [16] and prostate cancer [17]. However, the role of 17β-estradiol in the EMT of endometrial adenocarcinoma has not been studied.

The potential role of metformin in treating endometrial cancer has been explored in a number of studies [18-20]. Metformin likely exerts its antitumorigenic effects through indirect mechanisms by increasing insulin sensitivity, inhibiting liver gluconeogenesis, and reducing hyperglycemia and insulin levels [21], and direct mechanisms involving activating AMP-activated protein kinase (AMPK), followed by inhibition of the mammalian target of rapamycin (mTOR) pathway [22] and the attenuation of extracellular signal-regulated kinase (ERK) signaling [23]. In addition, metformin has been reported to repress the EMT in breast cancer [24] and lung cancer [25].

In the current study, we investigated the role of 17β-estradiol in the EMT and the effect of metformin on the EMT and 17β-estradiol-induced EMT in endometrial adenocarcinoma cells. In addition, we explored the signaling pathways that might be involved in this process.

## RESULTS

### Metformin inhibits the 17β-estradiol-induced proliferation, migration, and invasion of endometrial adenocarcinoma cells

**Figure 1:**
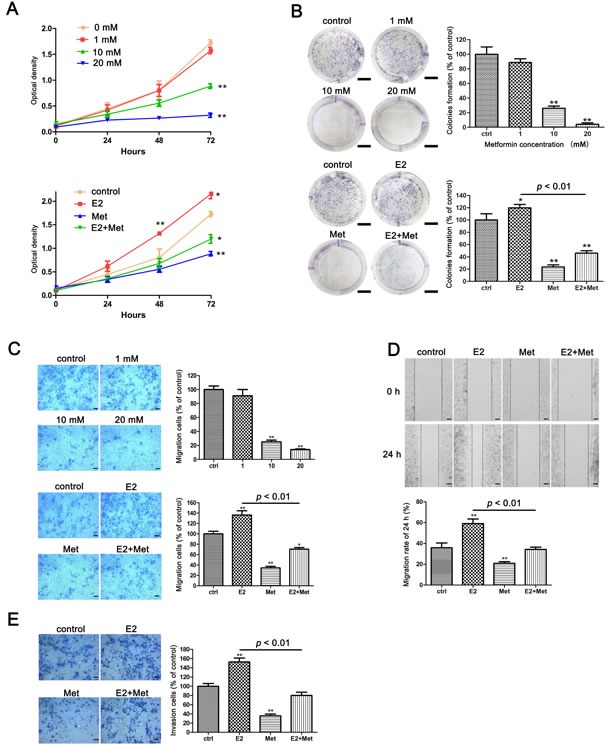
Metformin inhibits E2-induced proliferation, migration, and invasion in Ishikawa cells **A.** Ishikawa cells were treated with metformin (0, 1, 10, and 20 mM) (upper) and E2 with or without metformin (lower), cell numbers were measured by CCK-8 assays at indicated times. **B.** Colony formation assays were used to measure the colonogenicity of Ishikawa cells after E2 or/and metformin treatment. The number of untreated cells was set as 100%. Scale bar: 1 cm. **C.** Transwell migration assays of Ishikawa cells after E2 and metformin treatment. Representative images were obtained at 100× magnification. Graphs show the number of migration cells for each treatment group (averaged across three random images). Scale bar: 50 μm. **D.** Wound-healing assays of Ishikawa cells. Representative images were obtained at 40× magnification. Graphs show the relative migration distance after 24 h incubation. Scale bar: 125 μm. **E.** Transwell invasion assays of Ishikawa cells. Representative images were obtained at 100× magnification. Graphs show the number of invasion cells for each treatment group (averaged across three random images). Scale bar: 50 μm. Data represent the mean ± SD from one representative experiment of three independent experiments. E2: 17β-estradiol; Met: metformin. * *p* < 0.05, ** *p* < 0.01 *vs*. untreated cells.

**Figure 2:**
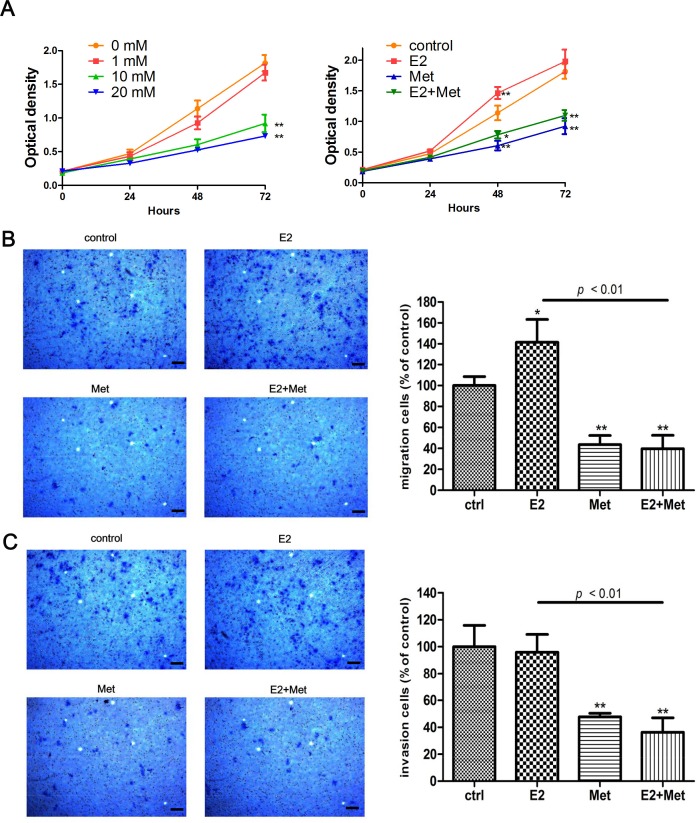
Metformin inhibits E2-induced proliferation and migration in KLE cells **A.** KLE cells were treated with metformin (0, 1, 10, and 20 mM) (left) and E2 with or without metformin (right), cell numbers were measured by CCK-8 assays at indicated times. Transwell migration assays **B.** and invasion assays **C.** of KLE cells after E2 or/and metformin treatment. Representative images were obtained at 100× magnification. Graphs show the number of migration or invasion cells for each treatment group (averaged across three random images). Scale bar: 50 μm. Data represent the mean ± SD from one representative experiment of three independent experiments. E2: 17β-estradiol; Met: metformin. * *p* < 0.05, ** *p* < 0.01 *vs*. untreated cells.

The Cell Counting Kit-8 (CCK-8) assays were performed to determine the effects of metformin and 17β-estradiol on the proliferation of endometrial adenocarcinoma cell lines, Ishikawa and KLE cells. Metformin inhibited the proliferation of both cell lines in a dose-dependent manner (Figures1A, 2A). Treatment with 17β-estradiol significantly increased the proliferation of both cell lines at 48 h and 72 h, which was abolished by the addition of metformin (Figures 1A, 2A). Similar data were obtained in the colony formation assays of Ishikawa cells (Figure 1B).

The migration and invasion ability of cells were determined using transwell assays and wound healing assays. Metformin inhibited the migration of Ishikawa cells in a dose-dependent manner. At concentrations of 10 and 20 mM, metformin significantly decreased the migration of Ishikawa cells (Figure 1C). 17β-estradiol significantly increased cell migration in both Ishikawa and KLE cell lines at 24 h, which was abolished by the addition of metformin (Figures 1C, 2B). Similar data were obtained from the wound healing assays of Ishikawa cells (Figure 1D). Moreover, 17β-estradiol significantly increased the invasion of Ishikawa cells at 36 h, which was also abolished by the addition of metformin (Figure 1E). However, 17β-estradiol showed no effect on the invasion of KLE cells (Figure 2C).

### Metformin antagonizes the anti-apoptosis effect of 17β-estradiol on endometrial adenocarcinoma cells

**Figure 3:**
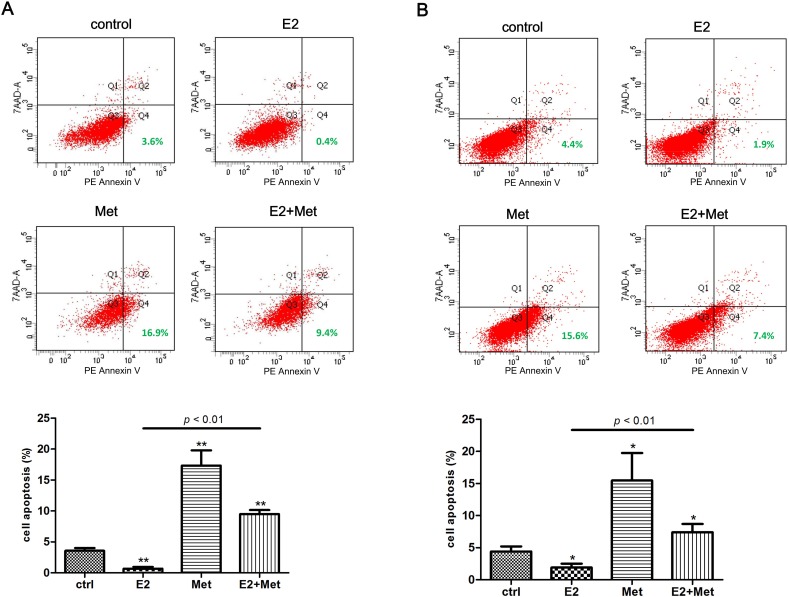
Metformin antagonizes 17β-estradiol-induced anti-apoptosis effect in endometrial adenocarcinoma cells Ishikawa cells **A.** and KLE cells **B.** were treated with metformin, E2, or both for 48 h. Cells were harvested and stained with PE Annexin V and 7-AAD, and cell apoptosis was analyzed using flow cytometry. Representative images were showed. The percentages of PE Annexin V ^+^ /7-AAD ^−^ cells (early apoptosis) were plotted. The data are presented as the mean ± SD of three replicates per group. E2: 17β-estradiol; Met: metformin. * *p* < 0.05, ** *p* < 0.01 *vs*. untreated cells.

Flow cytometry assays were performed to detect the effect of metformin and 17β-estradiol on the apoptosis of Ishikawa and KLE cell lines. In cells treated with 17β-estradiol, the PE Annexin V^+^/7-AAD^−^ (early apoptosis) subpopulation was significantly decreased compared to untreated cells in both cell lines (Figure 3A, 3B). In cells treated with 10 mM metformin, the early apoptosis rate increased significantly. Furthermore, the addition of metformin significantly abolished the 17β-estradiol-induced anti-apoptosis effects in both cell lines.

### Metformin reverses the EMT in endometrial adenocarcinoma cells involving the regulation of βKlotho, ERK1/2 and AMPKα signaling pathways

**Figure 4:**
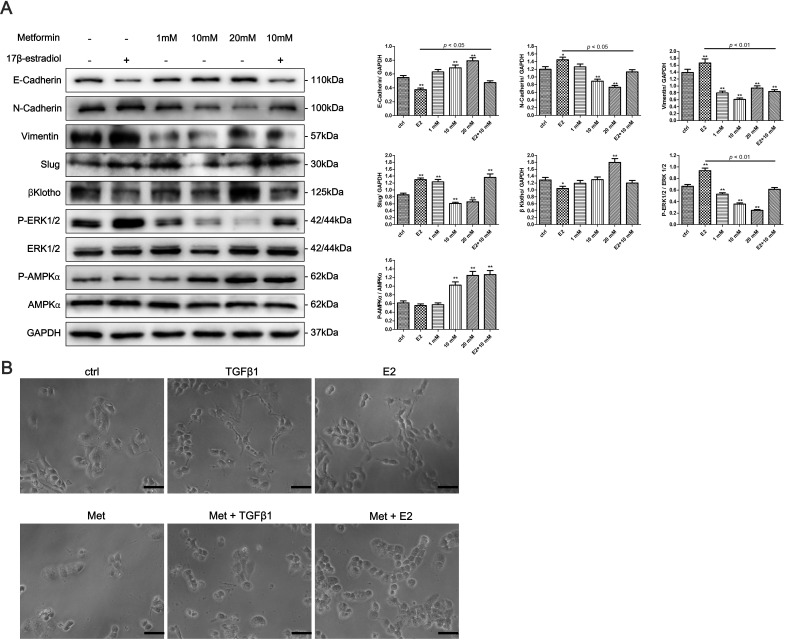
Metformin reverses 17β-estradiol-induced EMT in Ishikawa cells involving βKlotho expression, ERK1/2 and AMPKα signaling pathways **A.** Cells were treated with E2, metformin (1, 10, and 20 mM), or both agents for 48h. The protein expression levels of E-cadherin, N-cadherin, Vimentin, Slug, βKlotho, P-ERK1/2, ERK1/2, P-AMPKα, AMPKα, and GAPDH were presented by Western blot. GAPDH was used as a loading control. Expression ratios of E-cadherin to GAPDH, N-cadherin to GAPDH, Vimentin to GAPDH, Slug to GAPDH, P-ERK1/2 to ERK1/2, and P-AMPKα to AMPKα were analyzed. **B.** The morphology of Ishikawa cells treated with TGF-β1, E2, metformin, combination of TGF-β1 and metformin, and combination of E2 and metformin for 48h. The cells were observed using phase contrast microscopy at 200× magnification. Scale bar: 50 μm. The data are presented as the mean ± SD of three replicates per group. E2: 17β-estradiol; Met: metformin. *p < 0.05, ** *p* < 0.01 *vs*. untreated cells.

**Figure 5:**
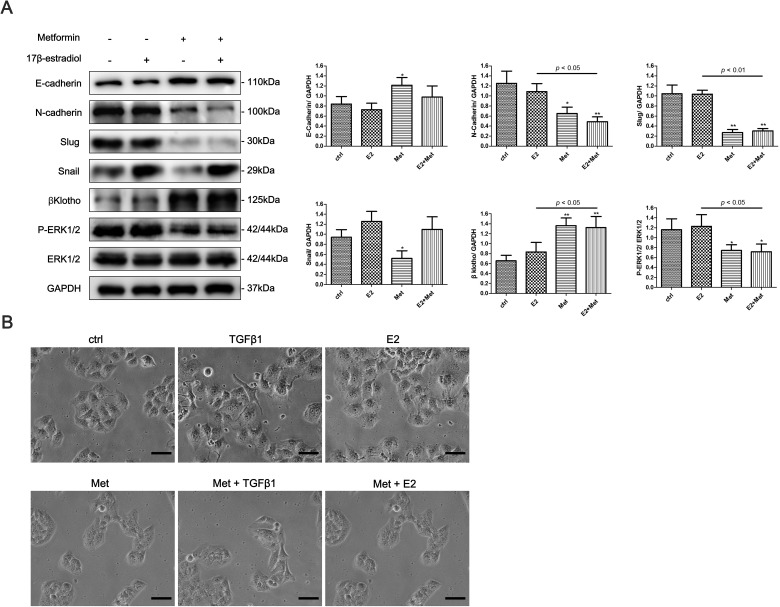
Metformin reverses the EMT in KLE cells *via* regulating βKlotho expression and ERK1/2 signaling pathway **A.** KLE Cells were treated with E2, 10mM metformin, or combination of the two agents for 48h. The protein expression levels of E-cadherin, N-cadherin, Slug, Snail, βKlotho, P-ERK1/2, ERK1/2, and GAPDH were presented by Western blot. GAPDH was used as a loading control. Expression ratios of E-cadherin to GAPDH, N-cadherin to GAPDH, Slug to GAPDH, Snail to GAPDH, and P-ERK1/2 to ERK1/2 were analyzed. **B.** The morphology of KLE cells treated with TGF-β1, E2, metformin, combination of TGF-β1 and metformin, and combination of E2 and metformin for 48h. The cells were observed using phase contrast microscopy at 200× magnification. Scale bar: 50 μm. The data are presented as the mean ± SD of three replicates per group. E2: 17β-estradiol; Met: metformin. *p < 0.05, ** *p* < 0.01 *vs*. untreated cells.

To determine whether 17β-estradiol and metformin are involved in the EMT regulation of endometrial cancer, we examined the expression of EMT-related markers in Ishikawa and KLE cell lines using western blot analysis. 17β-estradiol significantly decreased the expression of E-cadherin and increased the expressions of N-cadherin, Vimentin, and Slug in Ishikawa cells (Figure 4A). In addition, metformin reversed 17β-estradiol-induced EMT-markers expression by repressing N-cadherin and Vimentin expressions and restoring E-cadherin expression (Figure 4A). However, no significant effect was noted on the expressions of E-cadherin, N-cadherin, Slug, and Snail after treatment with 17β-estradiol in KLE cells (Figure 5A). At concentration of 10 mM, metformin significantly increased the expression of E-cadherin and decreased the expressions of N-cadherin, Slug and Snail in KLE cells (Figure 5A).

Next, we explored the possible signaling pathways that may be involved. We found that 17β-estradiol significantly decreased the expression of βKlotho, a coreceptor of fibroblast growth factor receptor (FGFR) signaling, in Ishikawa cells but not in KLE cells (Figures 4A, 5A). Simultaneously, 17β-estradiol significantly induced the phosphorylation of ERK1/2 (Figure 4A), which is the main downstream signaling intermediate of FGF signaling. In contrast, 17β-estradiol showed no activation on the ERK1/2 signaling in KLE cells (Figure 5A). Moreover, metformin significantly increased the expression of βKlotho and decreased ERK1/2 phosphorylation in a dose-dependent manner in both Ishikawa cells and KLE cells (Figure 5A). Metformin significantly increased AMPKα phosphorylation in the Ishikawa cells in a dose-dependent manner (Figure 4A).

We next examined the effect of 17β-estradiol and metformin on the morphology of Ishikawa and KLE cell lines. The cells were treated with recombinant transforming growth factor-β1 (TGF-β1), which is known to play a major role in inducing EMT. As expected, after stimulation with 0.78 nM of recombinant TGF-β1 for 48 h, both Ishikawa and KLE cells became scattered, acquired a spindle-shaped morphology, and lost cell-cell contacts, which are characteristics of a mesenchymal-like morphology (Figures 4B, 5B). 17β-estradiol exhibited similar effects as TGF-β1 in Ishikawa cells but not in KLE cells. Treatment with 10 mM metformin for 48 h abolished the TGF-β1 or 17β-estradiol-induced morphological changes in Ishikawa and KLE cell lines.

### Loss of βKlotho expression is present in human endometrial adenocarcinomas

**Figure 6:**
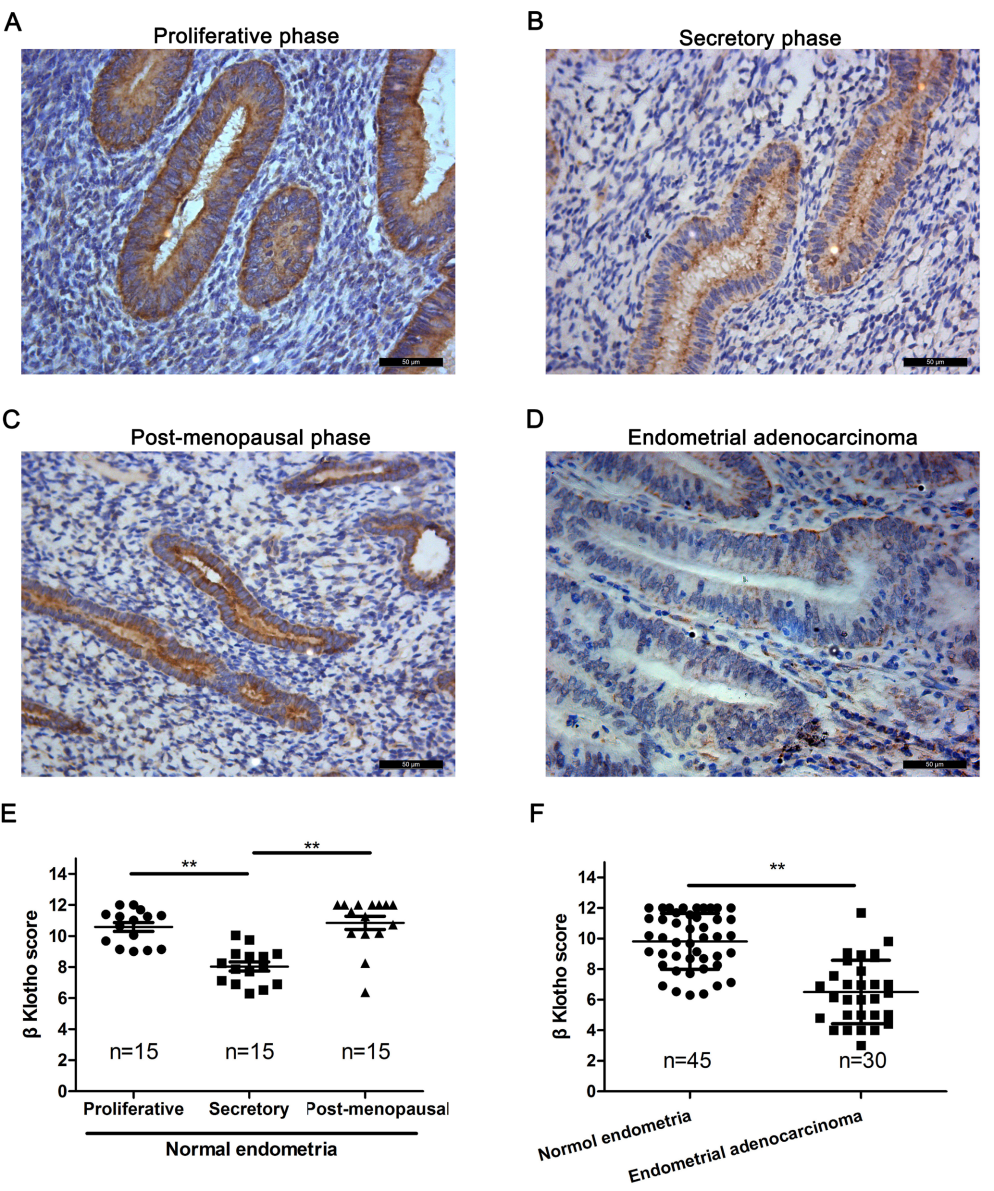
βKlotho expression is decreased in human endometrial adenocarcinomas The expression of βKlotho was shown by immunohistochemical analysis. **A.**. βKlotho expression in post-menopausal endometria. **B.** βKlotho expression in endometria of seretory phase. **C.** βKlotho expression in endometria of proliferative phase. **D.**. βKlotho expression in endometrial adenocarcinomas. **E.** The immunohistochemical score of βKlotho were calculated in proliferative phase (*n* = 15), secretory phase (*n* = 15) and post-menopausal phase of endometria (*n* = 15). **F.** The immunohistochemical score of βKlotho were calculated in normal endometria (*n* = 45) and endometrial adenocarcinomas (*n* = 30). Data was shown as the mean ± SD. Each experiment was performed in duplicate or triplicate. Scale bar: 50μm. ** *p* < 0.01.

The expression of βKlotho in human endometrial adenocarcinomas was determined by immunohistochemistry analysis. Normal endometria exhibited strongly positive βKlotho immunostaining (Figure 6A, 6B, 6C), and the staining was generally restricted to the cytoplasm and cytomembrane of epithelial cells. The βKlotho immunostaining was significantly stronger in the endometria of proliferative phase compared with those of secretory phase (Figure 6E). The βKlotho immunostaining in the endometria of post-menopausal phase was also stronger than those of secretory phase (Figure 6E). No significant difference was observed between the endometria of proliferative phase and post-menopausal phase (Figure 6E). The βKlotho immunostaining was significantly decreased in endometrial adenocarcinomas compared with normal endometria (Figure 6D, 6F).

### βKlotho expression inhibits 17β-estradiol-induced proliferation and the EMT by inhibiting ERK1/2 signaling pathway in endometrial adenocarcinoma cells

**Figure 7:**
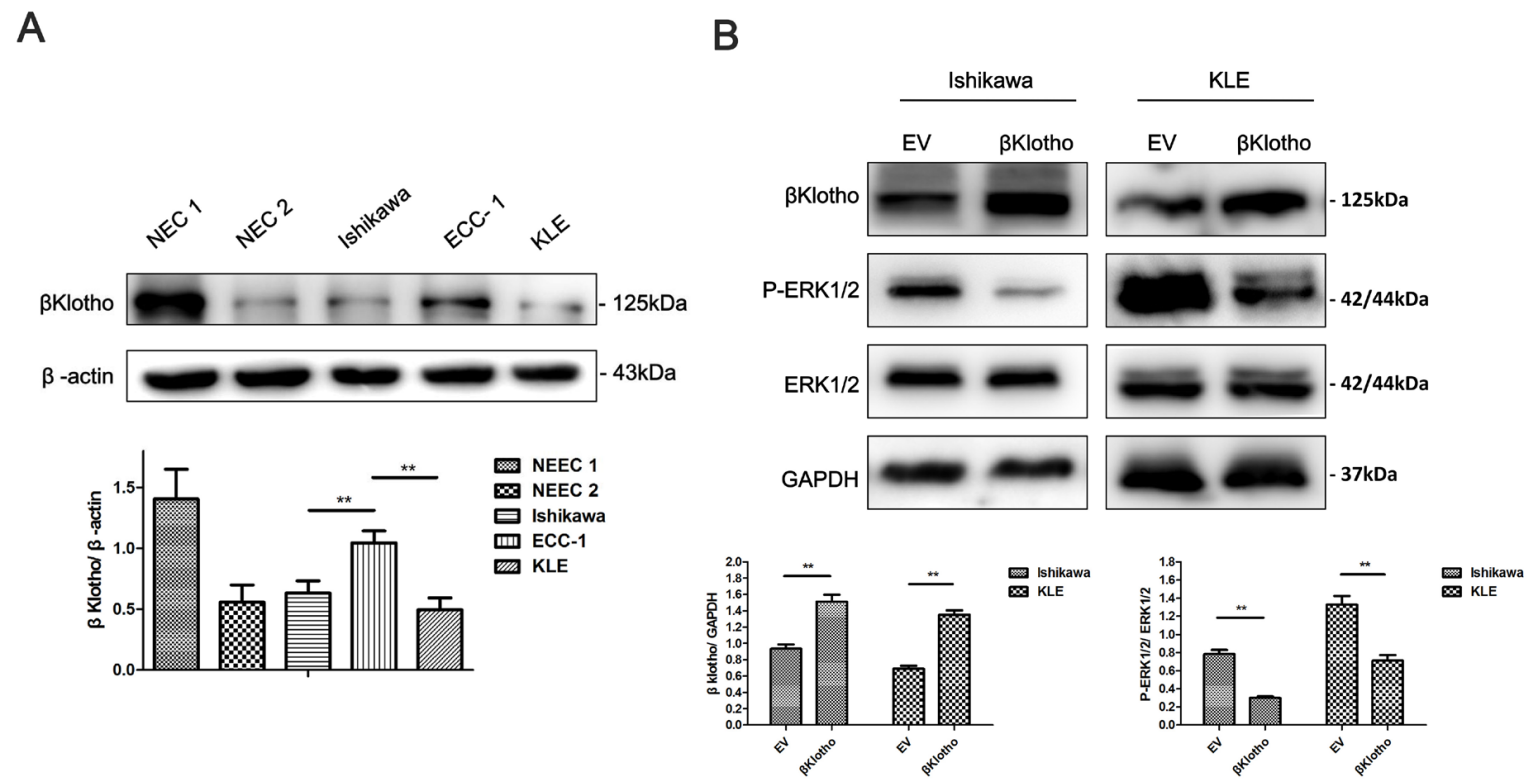
βKlotho expression inhibits ERK1/2 signaling pathway in endometrial cancer cells **A.** The protein expression levels of βKlotho in normal endometrial cells (NEC1 and NEC2), Ishikawa cells, ECC-1 cells and KLE cells were presented by Western blot. β-actin was used as a loading control. **B.** Western blot analysis of βKlotho, P-ERK1/2, and ERK1/2 in Ishikawa and KLE cells transfected with empty vector (EV) or βKlotho. GAPDH was used as a loading control. Expression ratios of βKlotho to GAPDH and P-ERK1/2 to ERK1/2 were analyzed. The data are presented as the mean ± SD of three replicates per group. ** *p* < 0.01.

**Figure 8:**
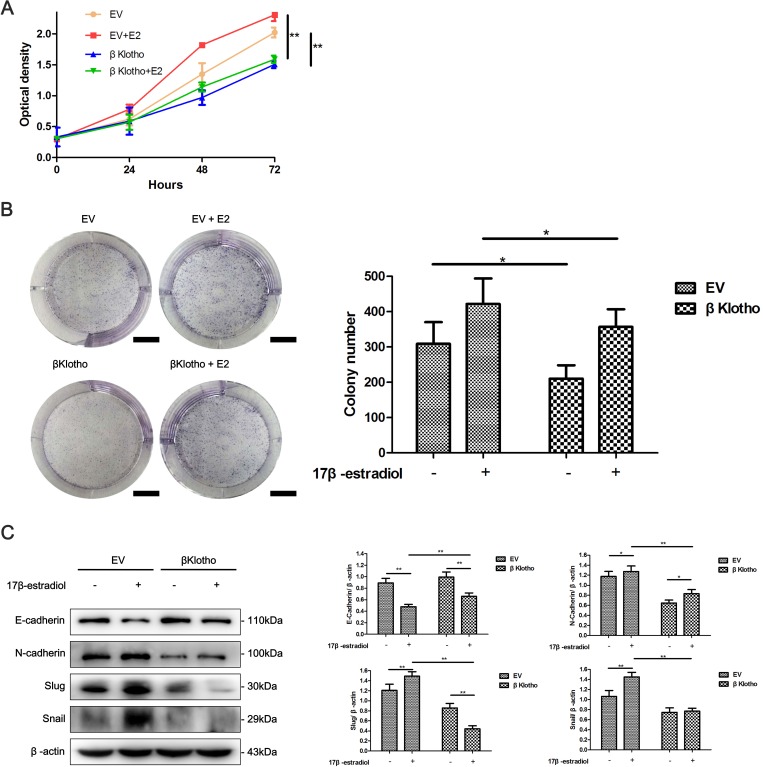
βKlotho expression inhibits 17β-estradiol-induced cell proliferation and EMT in Ishikawa cells **A.** Ishikawa cells transfected with either EV or βKlotho were treated with or without E2 for 48 h. Western blot was performed to detect the expression of E-cadherin, N-cadherin, Slug, and Snail. β-actin was used as a loading control. The EV- and βKlotho-transfected Ishikawa cells were treated with or without E2, CCK-8 assays **B.** and colony formation assays **C.** were performed at indicated times. Scale bar: 1 cm. The data are presented as the mean ± SD of three replicates per group. E2: 17β-estradiol. *p < 0.05, ** *p* < 0.01.

Stable clones were generated to determine the effect of βKlotho expression on the proliferation and EMT in endometrial adenocarcinoma cells. As shown in Figure 7A, βKlotho expression was determined in different endometrial epithelial cells using western blot analysis. Compared with endometrial adenocarcinoma cell line ECC-1 and normal endometrial cells (NEC) from two patients (named NEC 1 and NEC 2 respectively), Ishikawa and KLE cells exhibited lower βKlotho expression. Ishikawa and KLE cells were stably transfected with either the EV (empty vector) or βKlotho plasmid respectively, and the expression of βKlotho was confirmed by western blot analysis (Figure 7B). We found that βKlotho expression significantly decreased ERK1/2 phosphorylation in both cell lines (Figure 7B). Meanwhile, βKlotho expression significantly increased the expression of E-cadherin and decreased the expression of N-cadherin, Slug, and Snail (Figure 8A) in Ishikawa cell line. In addition, βKlotho expression also significantly abolished the 17β-estradiol-induced expression of N-cadherin, Slug, and Snail and restored E-cadherin expression (Figure 8A).

Using CCK-8 assays, we found that βKlotho expression significantly reduced the proliferation of Ishikawa cells and abolished 17β-estradiol-induced cell proliferation (Figure 8B). This inhibitory effect of βKlotho expression on cell proliferation was further demonstrated by colony formation assays (Figure 8C).

### βKlotho and metformin show synergetic effects on cell proliferation and the EMT in endometrial adenocarcinoma cells

**Figure 9:**
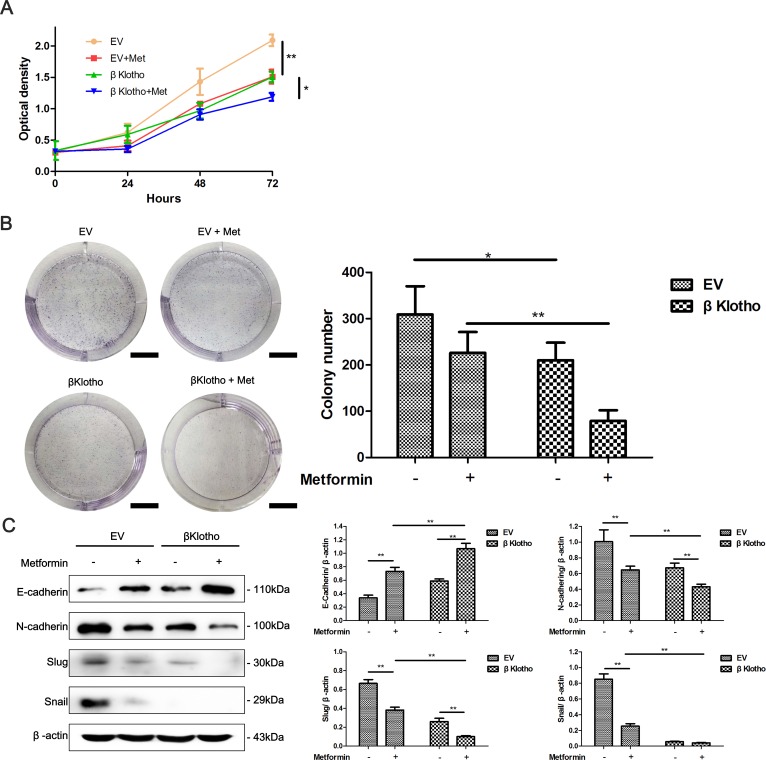
βKlotho expression and metformin show synergetic inhibitory effects on the proliferation and EMT in Ishikawa cells The EV and βKlotho transfected Ishikawa cells were treated with or without 10 mM metformin, CCK-8 assays **A.** and colony formation assays **B.** were performed at indicated times. Scale bar: 1 cm. C. Ishikawa cells transfected with either EV or βKlotho were treated with or without 10 mM metformin for 48h. Western blot was performed to detect the expression of E-cadherin, N-cadherin, Slug, and Snail. β-actin was used as a loading control. The data are presented as the mean ± SD of three replicates per group. Met: metformin. *p < 0.05, ** *p* < 0.01.

We have demonstrated that both metformin and βKlotho expression exhibit anti-proliferation and anti-EMT effects in endometrial adenocarcinoma cells. We performed CCK-8 assays, colony formation assays, and western blot analysis to investigate whether combination of metformin and βKlotho treatments has synergetic effects. As expected, with metformin treatment, the βKlotho-transfected Ishikawa cells showed significantly decreased cell proliferation compared to the EV-transfected Ishikawa cells (Figure 9A, 9B). Furthermore, metformin treatment increased the expression of E-cadherin, and decreased the expression of N-cadherin and Slug in both the βKlotho- and EV-transfected Ishikawa cells (Figure 9C). With metformin treatment, the βKlotho-transfected Ishikawa cells showed higher E-cadherin expression and lower expression of N-cadherin, Slug, and Snail compared with EV-transfected Ishikawa cells (Figure 9C).

### Metformin inhibits the EMT in endometrial adenocarcinoma cells partly *via* stimulating AMPKα signaling pathway

**Figure 10:**
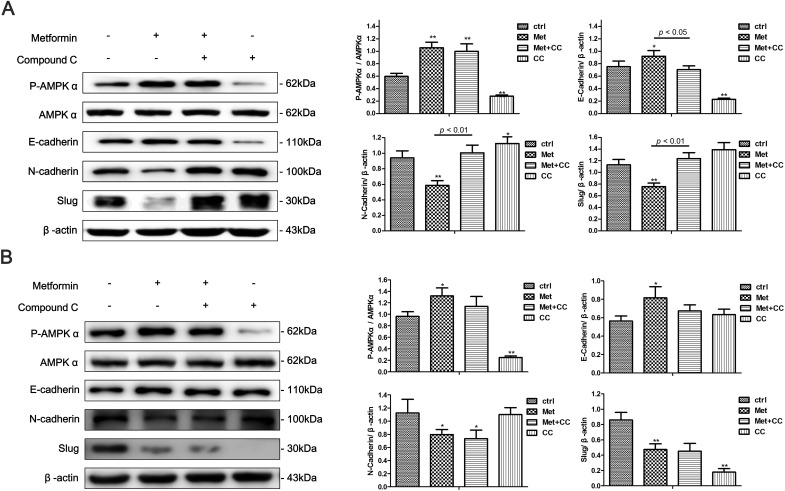
AMPKα inhibition partly blocks metformin-induced EMT reversal in endometrial adenocarcinoma cells Ishikawa cells **A.** and KLE cells **B.** were treated with 10 mM metformin, 10 μM Compound C or combination of the two agents for 48 h. Western blot was performed to detect the expression of P-AMPKα, AMPKα, E-cadherin, N-cadherin and Slug. β-actin was used as a loading control. Expression ratios of P-AMPKα to AMPKα, E-cadherin, N-cadherin, and Slug to β-actin were analyzed. The data are presented as the mean ± SD of three replicates per group. Met: metformin; CC: Compound C. * *p* < 0.05, ** *p* < 0.01 *vs* untreated cells.

AMPKα signaling is a well-studied downstream signaling pathway of metformin. Compound C, a specific AMPKα inhibitor, was added to cell cultures to investigate whether AMPKα signaling was involved in metformin-mediated EMT inhibition in Ishikawa and KLE cells. We found that the metformin treatment significantly increased AMPKα phosphorylation in both cells, which was abolished by the addition of Compound C (Figure 10A, 10B). Moreover, Compound C blocked the metformin-induced increase of E-cadherin expression and decrease of N-cadherin and Slug expression in the Ishikawa cells. However, the effect was not observed in KLE cell line (Figure 10A, 10B).

## DISCUSSION

**Figure 11:**
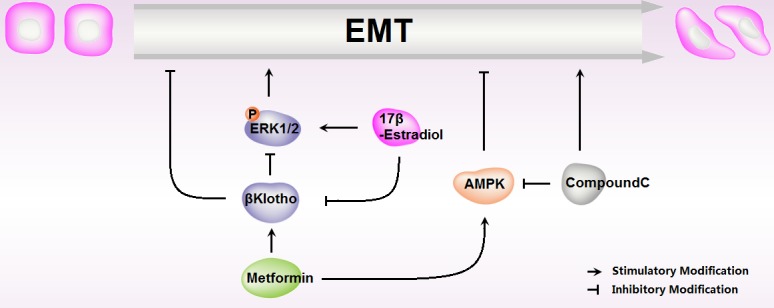
Schematic representation of metformin roles in 17β-estradiol-induced Epithelial-to-Mesenchymal Transition in endometrial adenocarcinoma cells

Diabetes, insulin resistance, and obesity are clear risk factors for the development of endometrial carcinomas [26, 27]. In recent years, metformin, the most widely used drug for type 2 diabetes [28], has been reported to decrease the incidence and progression of multiple human cancers [29, 30], and improve patients' overall survival rate, including endometrial carcinomas [31]. In the current study, we found that metformin reduced cell proliferation and EMT, abolished 17β-estradiol-induced cell proliferation and EMT in endometrial adenocarcinoma cells. In addition, βKlotho-mediated ERK1/2 signaling suppression and AMPKα signaling activation were involved in this effect (Figure 11).

Recent studies have reported that 17β-estradiol can induce the EMT in prostate cancer [32], breast cancer [33], and ovarian cancer [34]. Chen et al. found that the estrogen-induced EMT plays a crucial role in the development of adenomyosis [35]. Continuous exposure of the endometrium to estrogens is presumed to be the primary cause of endometrial carcinomas [36]. In the current study, we showed that 17β-estradiol treatment significantly promoted cell proliferation and migration in both ER-positive and ER-negative endometrial carcinoma cells. More importantly, we demonstrated that 17β-estradiol exposure led to the acquisition of both phenotypic and molecular attributes in the ER positive Ishikawa cells that are typical of the EMT. However, 17β-estradiol treatment did not induce EMT in ER negative KLE cells. The data indicated that endometrial carcinoma cells undergo EMT upon 17β-estradiol stimulation involving estrogen/ER signaling. Except estrogen/ER signaling, body mass index (BMI) has crucial effect on endometrial cancer. It was reported that high BMI [37] and low ER level [38] are two important factors associated with poor outcome of endometrial cancer. However, the literatures concerning association between BMI and ER status in endometrial cancer were rare, which needs further elucidated.

Metformin has been reported to influence EMT by acting upon pathways that inhibit TGFβ- [39] and interleukin-6-induced [25] EMT. Here, we found that metformin inhibited the EMT and 17β-estradiol-induced EMT in both ER-positive and ER-negative endometrial carcinoma cells. To our knowledge, this is the first study showing that metformin could inhibit the 17β-estradiol-induced EMT in tumor cells. Several studies have indicated that metformin decreases cancer cell viability by inducing apoptosis in endometrial cancer [20, 40]. In the current study, we observed that metformin not only induced apoptosis in endometrial cancer cells, but also reversed the anti-apoptosis effect of 17β-estradiol. The data emphasizes the potential therapeutic implication of metformin for estrogen-sensitive endometrial carcinomas.

ERK1/2 are considered as the classic mitogen-activated protein kinases (MAPKs). ERK1/2 activation causes the phosphorylation of nuclear transcription factors and eventually leads to a series of responses in the target cells, including cell differentiation, proliferation, and death [41]. Recent studies have showed that ERK1/2phosphorylationis involved in the EMT in cancer cells [42, 43]. In previous studies, we proved that MAPK activation can significantly promote the growth of endometrial carcinoma cells [44]. Here, we found that metformin led to a reduction of ERK1/2phosphorylationin endometrial carcinoma cells in a dose-dependent manner, indicating that the ERK1/2 signaling pathway may be involved in the metformin-induced reversal of EMT in endometrial carcinoma cells.

βKlothois a type I membrane protein that belongs to the Klotho family. The extracellular domain ofβKlotho is homologous to members of glycosidase family 1, but lacks glucosidase enzymatic activities [45]. βKlothois involved in the control of bile acid and lipid and glucose metabolism [46]. Itusually forms a complex with fibroblast growth factor receptors (FGFRs) and functions as a coreceptor for FGFs to activate FGFR signaling [47]. Evidence has linked carcinogenesis and EMT in a range of tissue types with the dysregulation FGFR signaling. It was recently reported that FGFR aberrations were found in 7.1% of cancers [48], while FGFR2 activating mutations were found in 10-16% of primary endometrial cancers [49].

In the current study, for the first time, we evaluated βKlotho expression in normal and cancerous endometrial tissues. The expression of βKlotho was hormone-dependent in normal endometria of reproductive aged women and maintained a higher level in the endometria of post-menopausal phase. However, βKlothoexpression was significantly decreased inhuman endometrial adenocarcinomas, which usually occurred in menopausal and perimenopausal women. In addition, 17β-estradiol decreased βKlotho expression in ER positive endometrial carcinoma cells. Plasmid-driven βKlotho expression downregulated the activity of ERK1/2 signaling, inhibited cell proliferation, and reversed the EMT in endometrial carcinoma cells. These data indicated that βKlotho is involved in the pathogenesis of endometrial carcinomas, especially that of estrogen-dependent type I endometrial carcinomas.

Since metformin was proved to inhibit 17β-estradiol-related cell proliferation and EMT, we further explored the effect of metformin on βKlotho expression. We found that metformin increased βKlotho expression in both ER-positive and ER-negative cancer cells. Additionally, βKlotho expression and metformin treatment exhibited synergetic effects on the inhibition of cell growth, migration, invasion in endometrial cancer cell. These data suggested that βKlotho-related ERK1/2 signaling pathway was involved in the inhibitory effect of metformin on the EMT and cell proliferation in endometrial carcinoma cells.

AMPKα is a well-known downstream molecule of metformin, and emerging evidence has indicated that metformin inhibits cancer cell proliferation by activating AMPKα [50]. Here, we found that AMPKα was activated in the metformin-treated endometrial adenocarcinoma cells. AMPKα inhibition by Compound C prevented metformin-induced EMT inhibition in Ishikawa cells, but not in KLE cells. These data indicated that the AMPKα signaling pathway may play a partial role in the EMT of endometrial carcinomas.

Collectively, the present study showed that metformin inhibits cell proliferation and the EMT, as well as the 17β-estradiol-induced proliferation and EMT in endometrial adenocarcinoma cells through βKlotho-related ERK1/2 signaling and AMPKα signaling. The data reinforce the potential benefit of metformin in treating endometrial cancer and provides novel mechanistic insights into its antitumor effects.

## MATERIALS AND METHODS

### Reagents and antibodies

Metformin, 17β-estradiol and Compound C were purchased from Sigma-Aldrich (St. Louis, MO, USA). The anti-human E-cadherin, anti-human N-cadherin, anti-human Vimentin, and anti-human βKlotho primary antibodies were purchased from Abcam (Cambridge, MA, USA). The anti-human ERK1/2, anti-human phospho-ERK1/2 (Thr202/Tyr204), anti-human AMPKα, anti-human phospho-AMPKα (Thr172), anti-human Slug, and anti-human Snail primary antibodies were purchased from Cell Signaling Technology (Danvers, MA, USA). The anti-human β-actin and anti-human GAPDH primary antibodies and HRP-conjugated goat anti-rabbit and HRP-conjugated goat anti-mouse secondary antibodies were purchased from ZSGB-BIO (Beijing, China).

### Tissue collection and immunohistochemistry analysis

Normal endometrial biopsies from 45 women (proliferative phase: *n* = 15, secretory phase: *n* = 15, and post-menopausal phase: *n* = 15, mean age: 44.2 ± 6.7) and cancer tissues obtained from 30 patients with endometrial adenocarcinoma (mean age: 51.3 ± 8.7) were employed in the immunohistochemistry analysis. The diagnosis was confirmed by histological examination. None of the participants received any hormonal therapy during the 3 months prior to their operation. The study was approved by the Institutional Research Ethics Committees of Shandong Provincial Hospital affiliated to Shandong University, and written informed consent was obtained from all patients. The fresh tissues were washed with phosphate-buffered saline (PBS) and then fixed in 4% paraformaldehyde. After dehydration and paraffin embedding, the samples were cut into 5 μm sections and mounted onto glass slides. The deparaffinized, rehydrated sections were incubated with 3% H_2_O_2_ and then antigen retrieval was performed. After blocking, the sections were incubated overnight with the rabbit anti-human βKlotho primary antibody (diluted 1:100 in PBS) in a humid chamber at 4°C. An HRP-conjugated goat anti-rabbit IgG was used as the secondary antibody.

The immunohistochemical score was composed of two elements: the number of positive cells and the intensity of the color reaction [51]. In brief, the number of positive cells was quantified as follows: 0 (no stained cells), 1 (1-10% positively stained cells), 2 (11-50% positively stained cells), 3 (51-80% positively stained cells) and 4 (81-100% positively stained cells). The staining intensity was quantified as follows: 0 (negative), 1 (weak), 2 (moderate) and 3 (strong). The immunoreactivity was calculated by multiplying these two scores, generating an immunoreactivity score of 0-12. Two sections per sample were assessed by two pathologists who were blind to the clinical or pathologic data. The experiments were repeated in duplicate or triplicate.

### Cell cultures and treatments

The human endometrial adenocarcinoma cell lines (Ishikawa, KLE and ECC-1) were purchased from the American Type Culture Collection (ATCC, USA). The Ishikawa cells were maintained in DMEM medium, and KLE and ECC-1 cells were maintained in RPMI 1640 medium, supplemented with 10% fetal bovine serum (FBS) and 1% penicillin/streptomycin at 37°C in a humidified environment with 95% air and 5% CO_2_. The isolation and culture of the normal endometrial cells were performed according to previously published data [51]. Metformin was dissolved in PBS and stored at −20°C. 17β-estradiol was dissolved in ethanol at a stock concentration of 10 mM and stored at −80°C. Compound C, a specific AMPKα inhibitor, was dissolved in dimethylsulfoxide (DMSO) at a stock concentration of 6.25 mM and stored at 4°C. The cells were treated with 10 nM 17β-estradiol and 10 μM Compound C. Mock treatments with an identical volume of PBS, ethanol or DMSO were used as controls.

### Generation of the stably transfected cell clone

The Ishikawa and KLE cells were plated on 6-well plate and maintained in DMEM and RPMI 1640 medium, respectively, containing 10% FBS without antibiotics. The cells were transfected with 2μg of the GV230-CON083 plasmid (empty vector, EV) or 2 μg of the GV230-βKlotho plasmid per well using the Roche X-tremeGENE HP DNA Transfection Reagent according to the manufacturer's protocol. The plasmids were constructed by Genechem (Shanghai, China). Twenty-four hours after transfection, the cells were placed under Geneticin (G418-sulfate, Ishikawa at 500 mg/L and KLE at 400 mg/L, Gibco, Invitrogen) selection for 14 days. Individual colonies were removed by trypsinization and expanded. The G418-resistant Ishikawa and KLE clones were maintained in medium containing Geneticin of 250 mg/L and 200 mg/L, respectively.

### Transwell assays

The transwell assays were performed using 24-well plates with 8-μm pore size inserts (Corning Life Sciences, NY, USA) according to the manufacturer's instructions. The cells were treated with various agents at the indicated concentration for 48 h before they were seeded into the inserts. In the migration assay, the cells (Ishikawa 5×10^4^ cells/well, and KLE 4×10^4^ cells/well) were added to the upper chamber in 200 μl of serum-free DMEM medium and allowed to migrate to the bottom compartment, which contained DMEM medium with 10% FBS, for 24 h. Then, the non-migrated cells were wiped off with a cotton swab.

For the invasion assay, Matrigel (1 mg/ml, BD Biosciences) was prepared in serum-free cold cell culture medium, placed in the upper chamber, and incubated for 5 h at 37°C. Next, the cells (10^5^ cells/well) were placed into the upper chamber of each insert in 200 μl of serum-free medium, and allowed to invade to the bottom compartment, which contained medium with 10% FBS, for 36 h. Then, the non-invaded cells were wiped off with a cotton swab.

For quantification, transwell filters were fixed in 4% paraformaldehyde for 15 min, stained with hematoxylin for 15 min, and mounted on a glass slide. The results are expressed as the number of cells migrated per field, as viewed under a microscope (× 100 magnification), and the numbers of cells in three randomly selected fields were counted. All experiments were performed three times.

### Wound healing assay

The cells were plated in 6-well culture plates in complete culture medium and allowed to grow to 90% confluence. A wound was created by scraping the well with a sterilized yellow pipette tip in the middle of the cell monolayer. After washing three times with PBS, the cells were cultured with fresh serum-free medium containing 17β-estradiol with or without the indicated concentration of metformin for 24 h. Subsequently, the ability of the cells to migrate into the cleared section was observed using a microscope. The migration rate was quantified by (scratch distance at 0 h - scratch distance at 24 h)/scratch distance at 0 h. Representative images were obtained at ×40 magnification. All experiments were repeated at least three times.

### Colony formation assay

The cells (3×10^3^/well) were plated on a 6-well plate in triplicate in 2 ml of medium containing 10% FBS and allowed to attach overnight. The next day, the medium was replaced with fresh medium containing 5% FBS with the indicated concentration of metformin, with or without 17β-estradiol. The cells were incubated at 37°C in a humidified atmosphere containing 95% air and 5% CO_2_ for 7 days. The culture medium was replaced every 3 days. Following incubation, the medium was removed. The colonies were fixed with 4% paraformaldehyde for 15 min, and then stained with hematoxylin for 15 min. The stained cells were rinsed three times with tap water to remove the excess dye. Each dish was then washed and dried. The colonies with diameter larger than 1.5 mm were counted. The experiment was performed three times.

### Cell proliferation assay

Cell viability was assessed using the Cell Counting Kit (CCK)-8 (Tongren, Shanghai, China). Briefly, the cells were plated on 96-well plates at a density of 4 ×10^3^ cells/well in 100 μL of medium. At 24 h after seeding, the indicated concentrations of metformin, with or without 17β-estradiol, were added to each well and the cells were cultured for an additional 24, 48, and 72 h, respectively. Ten μL of CCK-8 reagent was added to each well, and the plates were incubated at 37°C for 1 h. The optical density (OD) at 450 nm was measured in each well using a microplate reader. The experiments were repeated three times, and each assay was performed in triplicate.

### Apoptosis assay

The PE Annexin V Apoptosis Detection Kit I (BD Biosciences, USA) was used to measure apoptosis according to the manufacturer's instructions. Briefly, the cells were incubated with 10 mM metformin, with or without 17β-estradiol, for 48 h, collected, washed with cold PBS, gently resuspended in annexin V binding buffer, and incubated with PE Annexin V/7-AAD. Flow cytometry was performed using the CellQuest Pro software (BD Biosciences).

### Protein extraction and western blot analysis

The cells were harvested by centrifugation and washed with PBS. The cells were lysed in RIPA buffer containing protease inhibitors. Equal amounts of the protein lysates were electrophoretically separated on 10% SDS-PAGE gels and transferred to PVDF membranes. The membranes were blocked with 5% nonfat milk in Tris-buffered saline/0.1% Tween 20 for 1 h at room temperature and then incubated overnight at 4°C with the primary antibodies. After incubation with the secondary antibody for 1 h at room temperature, the protein bands were detected using the ECL detection system (BD Biosciences). β-actin or GAPDH were used as the loading controls.

### Statistical analysis

The statistical analyses were performed using SPSS 19.0 (SPSS Inc., Chicago, IL). The values are expressed as the means ± SD. The differences between the two groups were determined by the two-tailed Student's *t*-test. A *p* value < 0.05 was considered statistically significant.
